# Effect of Elevated Temperatures on the Mechanical Properties of a Direct Laser Deposited Ti-6Al-4V

**DOI:** 10.3390/ma14216432

**Published:** 2021-10-27

**Authors:** Sergei Ivanov, Marina Gushchina, Antoni Artinov, Maxim Khomutov, Evgenii Zemlyakov

**Affiliations:** 1 World-Class Research Center “Advanced Digital Technologies”, St. Petersburg State Marine Technical University, Lotsmanskaya 3, 190121 St. Petersburg, Russia; gushcina_mo@corp.smtu.ru (M.G.); e.zemlyakov@ltc.ru (E.Z.); 2 Bundesanstalt für Materialforschung und -Prüfung (BAM), Unter den Eichen 87, 12205 Berlin, Germany; Antoni.Artinov@bam.de; 3 Department of Physical Metallurgy of Non-Ferrous Metals, National University of Science and Technology “MISiS”, Leninsky Prospekt 4, 119049 Moscow, Russia; khomutov@misis.ru

**Keywords:** direct laser deposition, Ti-6Al-4V, mechanical properties, microstructure, stress relaxation, elevated temperatures

## Abstract

In the present work, the mechanical properties of the DLD-processed Ti-6Al-4V alloy were obtained by tensile tests performed at different temperatures, ranging from 20 °C to 800 °C. Thereby, the process conditions were close to the conditions used to produce large-sized structures using the DLD method, resulting in specimens having the same initial martensitic microstructure. According to the obtained stress curves, the yield strength decreases gradually by 40% when the temperature is increased to 500 °C. Similar behavior is observed for the tensile strength. However, further heating above 500 °C leads to a significant increase in the softening rate. It was found that the DLD-processed Ti-6Al-4V alloy had a Young’s modulus with higher thermal stability than conventionally processed alloys. At 500 °C, the Young’s modulus of the DLD alloy was 46% higher than that of the wrought alloy. The influence of the thermal history on the stress relaxation for the cases where 500 °C and 700 °C were the maximum temperatures was studied. It was revealed that stress relaxation processes are decisive for the formation of residual stresses at temperatures above 700 °C, which is especially important for small-sized parts produced by the DLD method. The coefficient of thermal expansion was investigated up to 1050 °C.

## 1. Introduction

Direct Laser Deposition (DLD) is one of the most widely utilized additive manufacturing (AM) technologies for the production of Ti-6Al-4V alloy parts. The uneven local heating of the buildup during DLD leads to significant stresses and distortion that affect the service properties and the shape of the final parts [1,2,3]. In the last, decade numerous models have been proposed for the simulation of these phenomena [4,5,6]. Thereby, the temperature dependence of the mechanical properties is a major factor influencing the accuracy of the simulation other than the assumptions of the mathematical model. Nowadays, it is a common practice to use the material properties of wrought Ti-6Al-4V alloys for the simulation of the DLD process. Mukherjee et al. [7] conducted a thermomechanical simulation of a DLD-processed Ti-6Al-4V-alloy by considering the material properties as being temperature dependent in the temperature range between 20 °C and 1600 °C. In their study, a combination of material properties derived from the fully lamellar wrought alloy [8] and the Ti-6Al-4V metal matrix composite [9] was used rather than the properties of the DLD-processed Ti-6Al-4V alloy. Denlinger and Michaleris [10] found a significant difference between the numerically predicted and experimentally measured distortion when using the mechanical properties of the wrought Ti-6Al-4V. Lu et al. [11] revealed significant scattering of the available data on the mechanical properties of the wrought Ti-6Al-4V alloy at elevated temperatures. Furthermore, a sensitivity analysis of the mechanical properties of Ti-6Al-4V showed that the distortion and the residual stresses strongly depend on the thermal expansion coefficient and less on the Young’s modulus and the elastic limit. They concluded that for the numerical analysis of the AM process, it is mandatory to use material properties that are specific to the particular manufacturing process. The present study is intended to fill the gap in the lack of data on the temperature dependence of the mechanical properties of DLD-processed Ti-6Al-4V alloys.

The poor mechanical properties of a commercially pure titanium hinder its application as a structural material. However, critical parts that require high strength and ductility, corrosion resistance in aggressive environments, heat resistance, etc., are made of titanium alloys. The Ti-6Al-4V alloy used in this study is a two-phase (α + β) alloy. Recognized as the most popular titanium alloy, Ti-6Al-4V occupies almost a half of the market share of titanium products used in the world today. The proportion of Al and V results in the material having attractive mechanical properties. Ti-6Al-4V contains 6 wt% Al, which stabilizes the α-phase of the hexagonal close-packed structure and 4 wt% V, which stabilizes the β-phase of the body-centered cubic structure. The two phases have different properties due to their structures, with α exhibiting greater strength yet lower ductility and formability [12]. The aluminum in the alloy increases the strength and heat-resistant properties, whereby vanadium increases not only the strength properties but also increases the ductility. It is well-known that two-phase titanium alloys have a lower sensitivity to hydrogen, e.g., hydrogen-induced cold cracking, compared to pseudo-α-alloys. Furthermore, they have good manufacturability and a relatively low tendency to undergo salt corrosion [13,14,15]. Titanium alloys have a good castability due to the short solidification interval of less than 50–70 °C [15,16]. Hereby, the chemical composition of the Ti-6Al-4V alloy utilized in casting does not differ from that of the wrought alloy [17]. The Ti-6Al-4V alloy utilized in additive manufacturing only has a slight difference in the content of carbon impurities from the wrought alloy [18].

It is a well-known fact that the mechanical and service properties of the alloy are determined by the microstructure. The high ductility and cyclic strength correspond to an equiaxed fine grain microstructure. On the other hand, the lamellar microstructure has a high fracture toughness and greater crack propagation resistance. Therefore, it can be said that the bimodal (duplex) microstructure offers an optimal combination of the mechanical properties of the wrought Ti-6Al-4V alloy. The control and optimization of the morphology of the α phase is one of the important issues in terms of the use of the alloy. Thermomechanical processing is a very useful method for improving the microstructure, e.g., controlling the size and the aspect ratio of the α lamellar phase, optimizing the phase ratio of the α to β phases, and controlling the morphology of the β phase [19,20]. The microstructure of the Ti-6Al-4V alloy obtained by direct laser deposition (DLD) depends strongly on the heat input and the inter-pass temperature [1,21], the variation of which results in a wide range of obtained mechanical properties. It is worth noting that the ductility of a DLD-processed alloy can fall to near zero, whereas the strength properties remain comparable to those of the wrought alloy [22].

**Figure 1:**
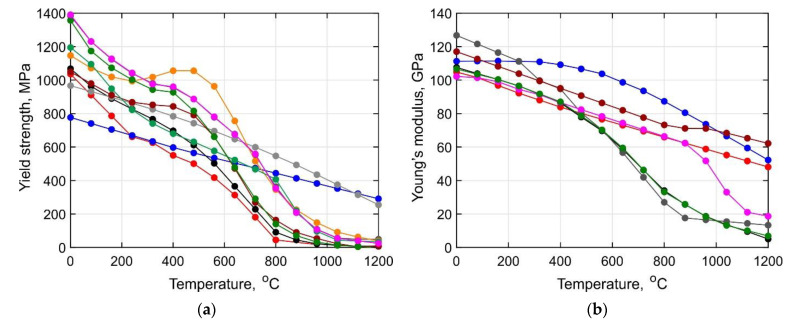
Temperature dependence of the (**a**) yield stress and (**b**) Young’s modulus according to [7,10,25,26,27,28,29,30,31,32,33].

The effect of the microstructure on the short-term strength of the Ti-6Al-4V alloy at elevated temperatures is similar to its effect on its strength at room temperature. The best combination of ductility, fracture toughness, heat resistance, and endurance is found in alloys with a 70–80% lamellar microstructure [15]. In [23], it was found that alloys with basket-weave microstructures exhibit the most obvious work hardening behavior and the highest strength during hot tensile deformation by temperatures of about 800 °C. The best ductility corresponds to alloys with an equiaxed microstructure. According to [24], the alloy with initial equiaxed microstructures also showed the highest ductility during tensile testing in the temperature range of 20–600 °C, while the material with initial full martensite microstructure showed better thermal strength. A critical analysis of the literature showed a significant spread in the experimental data of the short-term strength of the wrought alloy at elevated temperatures, as can be seen from Figure 1 [7,10,25,26,27,28,29,30,31,32,33]. This can be explained by the variation in the test conditions, the initial microstructure of the specimens, and the loading parameters.

It should be noted that there is practically no data available on the properties of additively manufactured Ti-6Al-4V alloy at elevated temperatures. In [34], an electron beam melting (EBM)-processed material showed a lower flow stress than the wrought alloy during a compression test in the temperature range of 1000–1200 °C. This can be attributed to the larger prior β-grain size and thickness of the α-plates in the EBM-processed alloy. In [35], the flow stress curves of the selective laser melting (SLM)- and direct energy deposition (DED)-processed and wrought alloys were compared in a compression test for temperatures ranging from 850 °C to 1100 °C. It was found that the presence of a percolating β-phase during the decomposition of martensite seems to be the reason for the reduced flow stress of the additively manufactured material compared to conventional wrought material with a lamellar microstructure. DED and SLM materials show a faster transformation to a globular microstructure compared to conventional wrought material. The temperature dependence of the tensile strength of the SLM-processed Ti-6Al-4V alloy in the temperature range between 20 °C and 550 °C was studied in [36]. SLM-processed Ti-6Al-4V alloy showed excellent ultimate tensile strength below 500 °C, which was 100 MPa higher than a solution-treated and aged Ti-6Al-4V alloy and 300 MPa higher than an annealed Ti-6Al-4V alloy. The effect of the strain rate and temperature on the mechanical properties of the DLD-processed alloy with a Widmanstätten microstructure was studied in [37], using a compression and tension test. However, the study lacks a description of the thermal history during the fabrication of the specimens. The presence of defects such as pores and a lack of fusion had a significant impact on the obtained results. The study presented in [38] compares the mechanical properties of a wrought alloy and a SLM-processed alloy in a compression test utilizing strain rates of 0.001–1 s^−1^ in the temperature range of 20–1000 °C. Both publications revealed that the anisotropy of the mechanical properties of the Ti-6Al-4V alloy obtained by various AM methods is insignificant.

The review presented above shows that most of the available material data for additively manufactured Ti-6Al-4V alloy are limited to the study of flow stresses at different strain rates in compression tests above 700 °C. These data are important for determining the parameters of the hot forging or stamping processes but are insufficient for the numerical simulation of stresses and distortion induced by the DLD. It should be noted that to the best of the authors knowledge, none of the publications contain data on the temperature dependence of the Young’s modulus of the Ti-6Al-4V alloy obtained with AM methods. Moreover, the majority of studies are devoted to the investigation of the material properties of SLM-processed alloys, which differ from those of the DLD-processed alloys.

In the present paper, the mechanical properties of a DLD-processed Ti-6Al-4V alloy were obtained through a tensile test performed for different temperatures ranging from 20 °C to 800 °C. The conditions used to obtain the test specimens were close to the conditions used in the manufacturing of large-sized structures by the DLD method. The influence of the thermal history on the stress relaxation for the case of 500 °C and 700 °C maximum temperatures was revealed. In addition, the temperature dependence of the coefficient of thermal expansion was obtained. The influence of the initial microstructure of the samples on the deformation and fractures at elevated temperatures was as well analyzed. An approximation of the measured tensile curves for given temperatures using a proposed fitting function was obtained and used to describe the hardening behavior during plastic deformation.

## 2. Materials and Methods

### 2.1. Specimens

**Figure 2:**
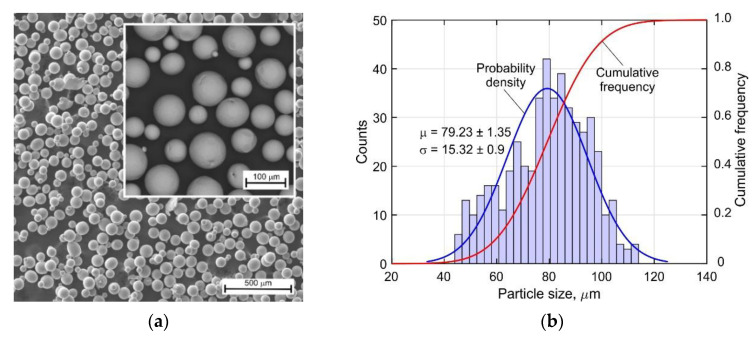
(**a**) Scanning electron micrograph of Ti-6Al-4V powder and (**b**) size distribution of the powder particles.

Almost all of data published on the mechanical properties of the DLD-processed Ti-6Al-4V alloy refer to samples obtained without or with very short dwell time between the deposited layers, leading to a significant overheating of the buildup. Therefore, in the present study, the tensile samples were machined from buildups with an inter-pass temperature in the range of 60–80 °C, which is typical for large-sized components. Note that the interpass temperature was controlled with type K thermocouples with a diameter of 0.5 mm. The DLD process parameters were as follows: a beam power of 1900 W; a beam diameter of 2.5 mm; a process speed of 20 mm s^−1^; a powder flow rate of 10.5 g min^−1^; and a gas flow rate of 25 L min^−1^. The specimens were made using an in-house robotic DLD machine that was developed at the St. Petersburg State Marine Technical University in St. Petersburg, Russia. The machine included a Fanuc 6500 5-axis industrial robot, a rotary table, and a processing head with a discrete coaxial powder feed. To prevent the oxidation of the specimens during the buildup, the sealed chamber of the machine was filled with argon. Hereby, the residual oxygen content in the chamber did not exceed 100 ppm. In total, 160 layers were deposited, having 12 mm in width, 0.8 mm in height, and 140 mm in length, and each layer consisted of seven passes. Spherical Ti-6Al-4V powder with a diameter of 45–90 µm, which was produced by a plasma rotating electrode method, was used for the buildups. The size distribution of the powder particles was unimodal with no visible non-metallic inclusions on the surface, as shown in Figure 2. The chemical composition was in accordance with the standard ASTM F136-02a [39].

### 2.2. Optical and Scanning Electron Microscopy

Optical metallography of etched microsamples was conducted using a Leica DMI8A microscope with a magnification of up to 1000 times. For the etching, Kroll’s reagent (1 mL HF + 2 mL HNO_3_ + 47 mL H_2_O) was used [40]. All metallographic cross-sections were taken from the middle of the buildup. The Vickers hardness was measured according to the ISO 6507 standard on an FM-310 hardness tester (Future Tech, Tokyo, Japan) with a load of 3 N. To determine the chemical composition and to analyze the fracture surface of the specimens after testing, a Tescan Mira3 scanning electron microscope (TESCAN, Brno, Czech Republic) with an Oxford AZtec console was used (Oxford Instruments NanoAnalysis, Wycombe, UK).

### 2.3. Tensile Tests at Elevated Temperatures

**Figure 3:**
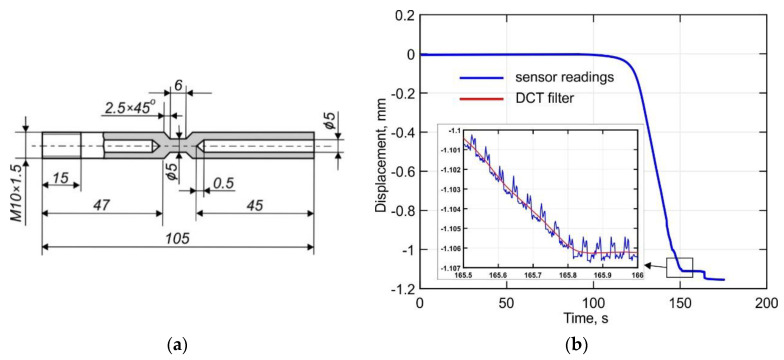
(**a**) A schematic of the Gleeble 3800 test specimen and (**b**) an example of experimental data processing using the DCT filter.

The mechanical properties of the DLD-processed Ti-6Al-4V alloy were obtained using a Gleeble 3800 metallurgical simulation system at the National University of Science and Technology MISiS in Moscow, Russia. The setup allows sequential tensile-compression deformation with a force of up to 10 t and simultaneous heating of the sample by direct electric current transmission to be performed. Depending on the specimen configuration and size, the heating and the cooling rate can reach up to 10,000 °C s^−1^ and 3000 °C s^−1^, respectively. The temperature field was controlled by the contact method using a type K thermocouple with a diameter of 0.25 mm that was fixed to the surface of the sample by discharge spot welding. A schematic of the specimen used for the uniaxial tension tests is shown in Figure 3. Thereby a heating rate of 10 °C s^−1^ and a strain rate of 3 mm min^−1^ were used. An externally mounted sensor was used for the precision recording of the transverse strain. The transverse strain was measured with a 500 Hz sampling rate in the central section of the specimen. The noise in the experimental data, which is shown in Figure 3b, significantly hampered their processing. Therefore, a robust discrete cosine transform (DCT) filter, which was implemented in the commercial software Matlab, was used to process the data [41,42].

An approximation of the measured tensile curves for given temperatures was performed to describe hardening behavior during plastic deformation. The following fitting function was proposed:

(1) $$\sigma (\varepsilon )=\frac{p_{1}\cdot \varepsilon^{2}+p_{2}\cdot \varepsilon +p_{3}}{\varepsilon +p_{3}}\cdot \sigma_{0.2},$$

where *σ* is the stress, *ε* is the strain, $\sigma_{0.2}$ is the yield strength, and *p*_1_, *p*_2_, *p*_3_ are the fitting coefficients.

**Figure 4:**
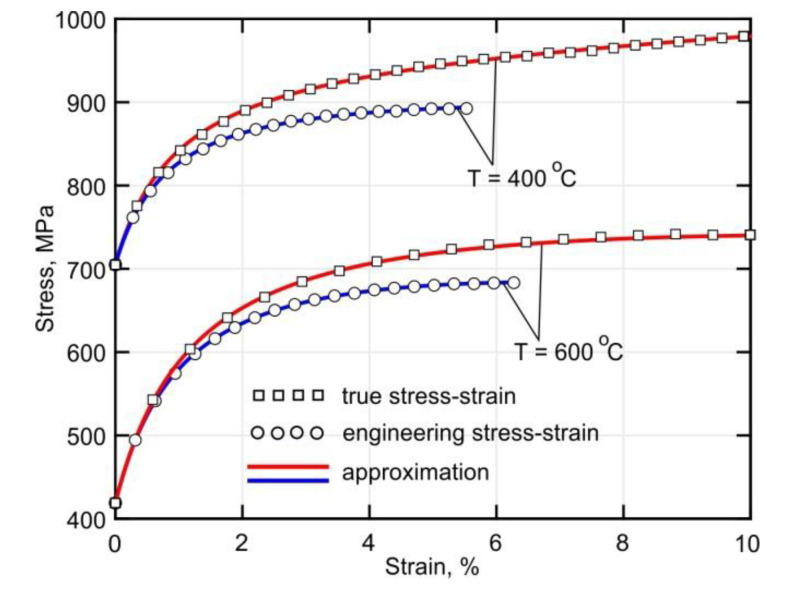
An example of the measured stress–strain curves and their approximation.

Note that a zero strain in Equation (1) corresponds to a stress level that is equal to the yield strength of the material. The fitting coefficients in Equation (1) were determined by the nonlinear least squares method [43]. Figure 4 shows an example of fitted experimental data. Note that for clarity, only a small part of the recorded experimental points is shown. It can be seen that the proposed fitting function agrees well with the experimental data. However, it should be noted that the fitting of the engineering stress–strain curves was conducted using the data included between the strain corresponding to the yield strength and the strain corresponding to the tensile strength.

### 2.4. Thermal Expansion Tests

**Figure 5:**
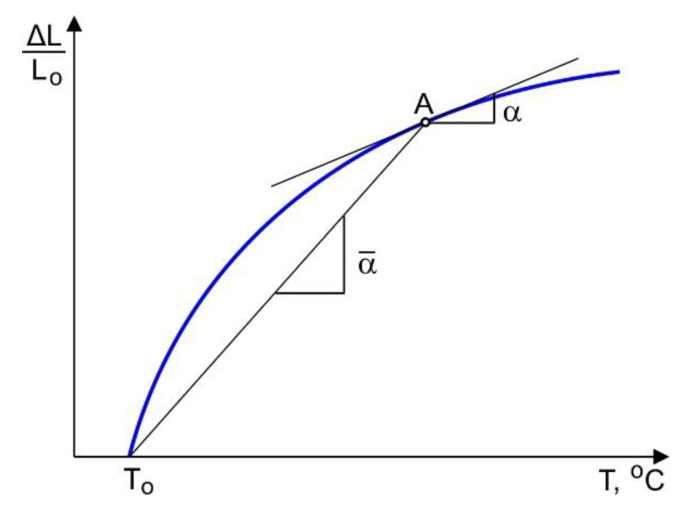
Determination of the coefficients of thermal expansion at point A.

A DIL 805 A/D quenching dilatometer test machine was used to determine the temperature dependent coefficient of thermal expansion (CTE). A cylindrical specimen with a 4 mm diameter and 10 mm length was inductively heated up to 1050 °C at a rate of 3 °C s^−1^. The tests were conducted in vacuum to prevent oxidation. After holding the sample at the maximum temperature for 20 min, the specimen was cooled at a rate of 0.94 °C s^−1^ by blowing it with helium. An instantaneous α and secant $\bar{\alpha }$ coefficient of thermal expansion were determined, according to Figure 5. Therefore, the following expressions were used:

- for instantaneous CTE:

(2) $$\alpha =\frac{\Delta L_{2}-\Delta L_{1}}{L_{o}}\cdot \frac{1}{T_{2}-T_{1}};$$

- for secant CTE:

(3) $$\bar{\alpha }=\frac{\Delta L(T)}{L_{o}}\cdot \frac{1}{\left(T-T_{o}\right)}.$$

The instantaneous CTE was determined by the first derivative of the experimental thermal strain curve with respect to the temperature. Note that an irregular experimental curve can cause significant high-frequency fluctuations of the calculated derivative values. Thus, to obtain a smooth curve of the instantaneous CTE, an experimental thermal strain curve was approximated by piecewise polynomials of 9th degree.

### 2.5. Stress Relaxation Tests

The visco-plastic behavior of DLD-processed Ti-6Al-4V alloy was measured using a uniaxial tensile test utilized in the Gleeble 3800 machine according to the method described in [44]. Each specimen was first heated to 500 °C or 700 °C and subsequently tensioned to a specified strain value. All samples were tensioned with a strain rate of 3 mm min^−1^ to a total strain of 2%, which is equal to a stress level of about 88% of the yield strength of the alloy at the corresponding temperature. In the next step, the applied strain was held constant, and the stress relaxation was measured as a function of time.

## 3. Results and Discussion

### 3.1. Microstructure of the DLD-Processed Ti-6Al-4V Alloy

**Figure 6:**
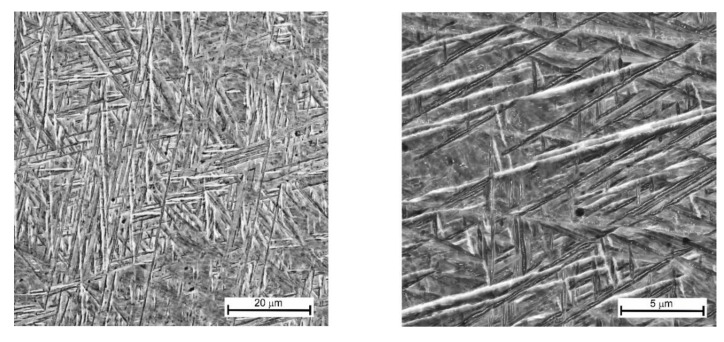
Microstructure of a Ti-6Al-4V buildup obtained by the DLD method.

The following factors affect the microstructure of the Ti-6Al-4V buildup: (1) a high crystallization rate of the deposited metal due to low interpass temperatures [45,46]; (2) multiple short-term irregular reheating phases from subsequent passes [47,48]; (3) epitaxial crystal growth [49,50]. The microstructure consisted of a lamellar α’-phase, as shown in Figure 6, and a small amount of residual β-phase in the form of thin interlayers [51,52,53]. The residual β-phase cannot be detected by optical or scanning electron microscopy due to its minor content. The presence of an α′-phase leads to an increase in the strength and a decrease in the ductility of the material. The nucleation of the α-phase initiates at the boundaries of the β-grains. The α-plates grow inside the grain until they meet plates growing from other boundaries during further cooling phases. As a result, colonies of unidirectional α-plates are formed in the grain. Cutting the lamellas of different colonies and grains at the different angles in the plane of the microsample results in a visible difference in the α-plate thickness. The thicknesses of such plates are close to each other. The average microhardness of the buildup alloy was 397 HV0.3.

### 3.2. Effect of the Temperature on the Fracture Behavior

**Figure 7:**
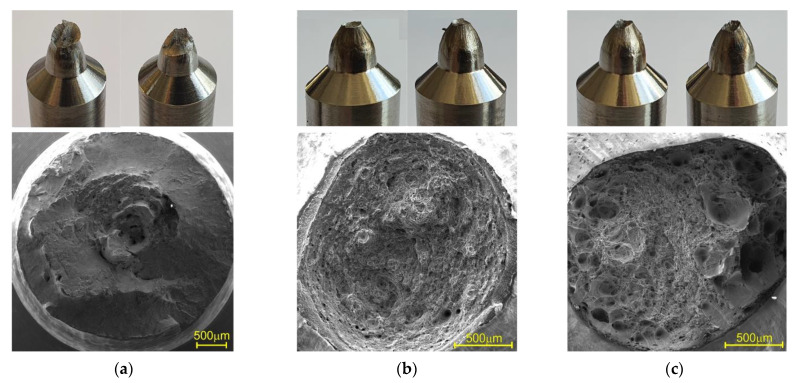
Fracture surface of specimens tested at (**a**) 200 °C, (**b**) 500 °C, and (**c**) 700 °C.

A macrograph of the fracture surface of the specimens tested at different temperatures is shown in Figure 7. All of the specimens had a cup-and-cone ductile fracture. A distinctive feature of the specimens tested at 200 °C, as shown Figure 7a, is the presence of two zones: a fibrous zone and a shear zone. The fibrous zone corresponds to the area of slow crack growth. It is located in the center of the fracture. The shear zone is an annular fracture zone that is adjacent to the free surface of the specimen. The extent of the shear zone decreases until it disappears completely as test temperature increases. This is clearly visible in the macrographs of the specimens tested at 500 °C and 700 °C, shown in Figure 7b,c. The higher test temperature corresponds to a significantly higher reduction of the area and the presence of large pores with a diameter of approximately 350 µm.

**Figure 8:**
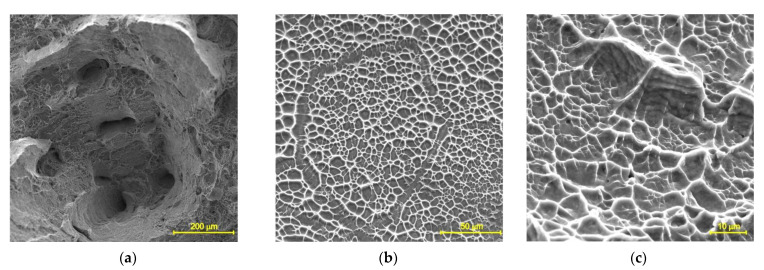
SEM fractograph of a specimen tested at 200 °C showing (**a**) the fibrous central zone and (**b**,**c**) the shear zone.

**Figure 9:**
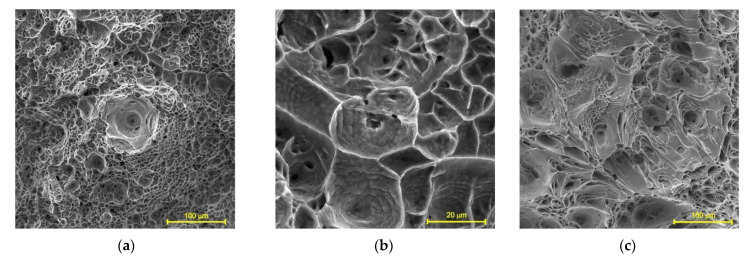
Fracture surface of specimen tested at (**a**,**b**) 500 °C and (**c**) 700 °C.

Deep dimples are clearly visible in the central region of the fracture surfaces of the specimens tested at 200 °C. A ductile local fracture is immediately initiated around those dimples, as seen in Figure 8a. The dimples are formed by the coalescence of micropores, which, in turn, grow and expand under a triaxial stress state [54,55]. Flat equiaxed dimples are clearly observed in the shear fracture zone shown in Figure 8b,c. They are formed due to the coalescence of micropores under the action of shear stresses. At higher test temperatures, the fracture is caused by the nucleation of the micropores at the grain boundaries, which are formed by a grain boundary slip, see Figure 9. The subsequent diffusion of vacancies or the development of local sliding leads to an enlargement of the pores. The larger dimples observed in Figure 9a correspond to triple grain boundary junctions. The small dimples seen in Figure 9b originate from the walls of the dislocation cells. At a test temperature of 700 °C, the pores are larger and deeper, as seen in Figure 9c. Note that the pores are elongated in the direction of plastic deformation.

### 3.3. Short-Term Mechanical Properties of the Ti-6Al-4V Alloy over a Wide Temperature Range

**Figure 10:**
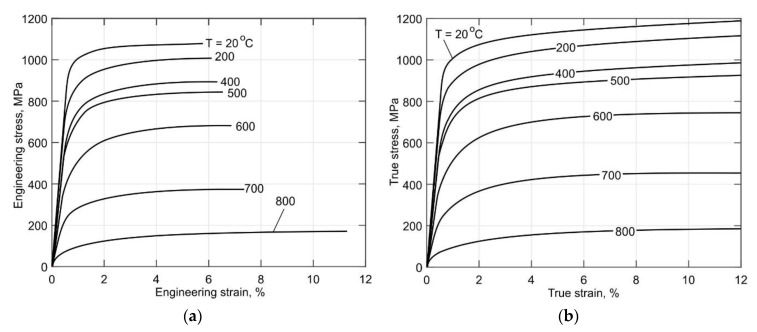
(**a**) Engineering and (**b**) true tensile curves of the DLD-processed Ti-6Al-4V alloy.

The experimentally obtained engineering and true tensile stress curves of the Ti-6Al-4V alloy for the temperature range between 20 °C and 800 °C are shown in Figure 10. The processing of the experimental data was conducted according to the procedure described in Section 2.3. It can be seen in Figure 10a that the total strain corresponding to the ultimate strength increases when the temperature increases. The ductility of the alloy increases significantly at temperatures above 700 °C. It should be noted that the ductility of the DLD-processed alloy at room temperature is comparable to that of the wrought alloy [13].

**Figure 11:**
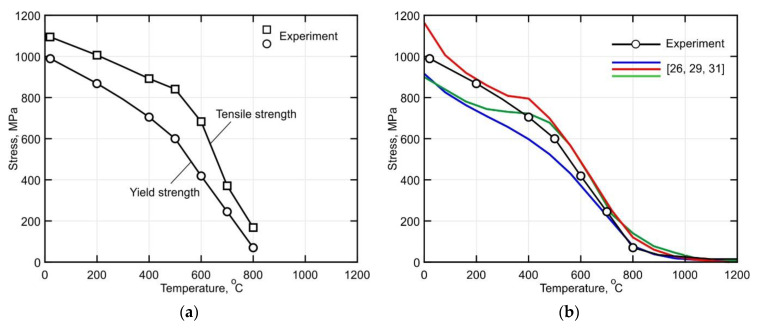
(**a**) Yield and tensile strength of the Ti-6Al-4V alloy as functions of temperature and (**b**) comparison of the obtained yield strength with published data from [26,29,31].

In Figure 11a the yield and tensile strength are plotted as functions of the temperature. It can be observed that the yield strength decreases gradually by approximately 40% as the temperature rises to 500 °C. A further increase of the temperature leads to a significant increase in the softening rate. This behavior is associated with the intensification of the diffusion-controlled decomposition of the metastable α’-phase and the grain boundary slip process [56]. Thus, the yield strength decreases almost linearly from 600 MPa to 70 MPa in the temperature range between 500 °C and 800 °C. According to the published data shown in Figure 11b, a further increase in the temperature leads to complete softening of the material. The green curve corresponds to the sample with the α’ -microstructure [29]. This shows a close correlation to the obtained curve for the DLD-processed alloy, especially at temperatures above 400 °C. The discrepancy between the curves in the temperature range of 20–400 °C can be explained by the differences in the size and the shape of the prior β-grain as well as by the morphology and the thickness of the α-plates [12]. On the other hand, the blue curve corresponds to the (α + β) microstructure [31] and shows lower yield strength values. However, its behavior is almost identical to that of the obtained curve for the DLD-processed alloy.

**Figure 12:**
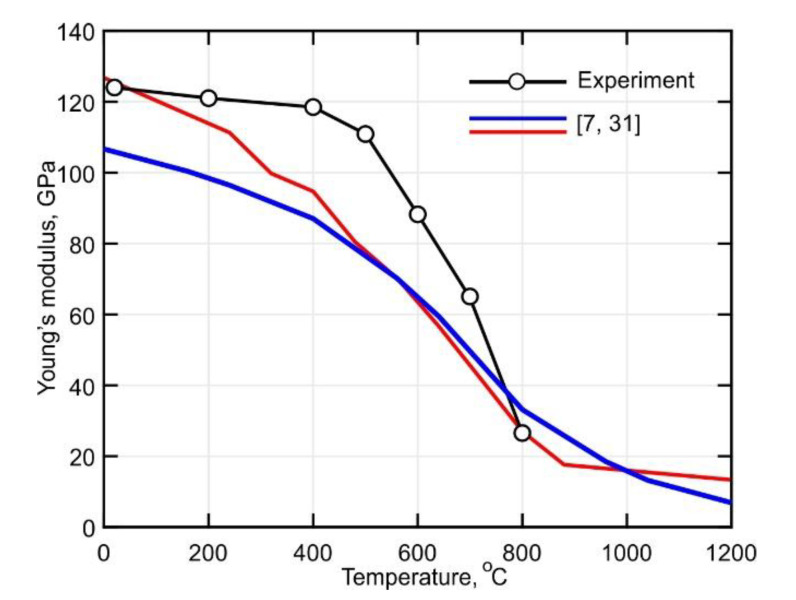
Comparison of the measured Young’s modulus of the Ti-6Al-4V alloy with published data from [7,31].

In Figure 12, the temperature-dependent Young’s modulus is shown. It is observed that the Young’s modulus remains almost unchanged for a temperature increase of up to 500 °C. However, it decreases sharply by approximately 70% from 109 GPa to 26 GPa upon further heating to 800 °C. A comparison of the obtained curves with previously published data shows a significant discrepancy. The blue curve corresponds to a sample with a bi-modal Widmanstätten microstructure obtained from a plate that was 12 mm thick. Note that the plates were treated by annealing for 6 h at 790 °C [31]. The DLD-processed alloy shows a Young’s modulus with greater thermal stability. At 500 °C the Young’s modulus of the alloy is about 46% higher than that of the wrought alloy.

An approximation of the obtained tensile curves for given temperatures using the proposed fitting function was performed according to the procedure described in Section 2.3. The obtained coefficients of the fitting function are given in Table 1. These data are of high importance for the numerical analysis of the residual stresses and distortion of additively manufactured parts.

### 3.4. Temperature Dependence of the Thermal Expansion Coefficient

**Figure 13:**
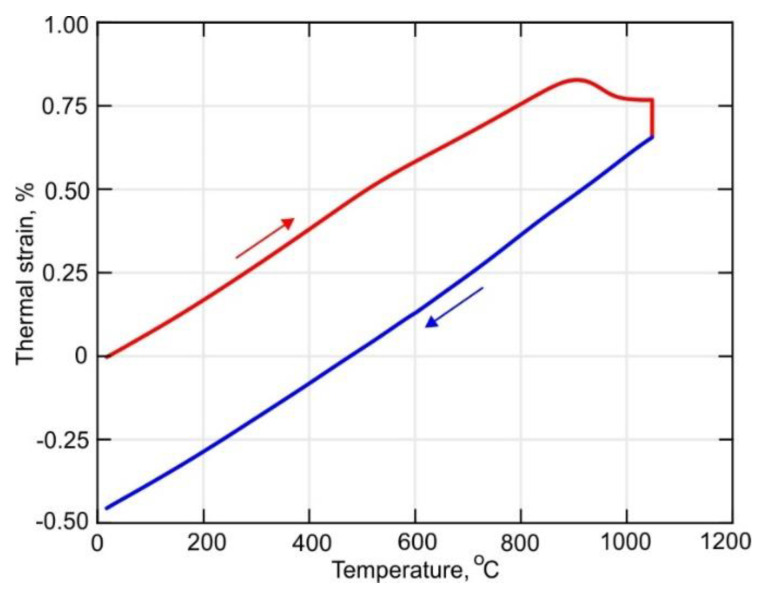
Temperature-dependent thermal strain curve of the DLD-processed Ti-6Al-4V alloy.

**Figure 14:**
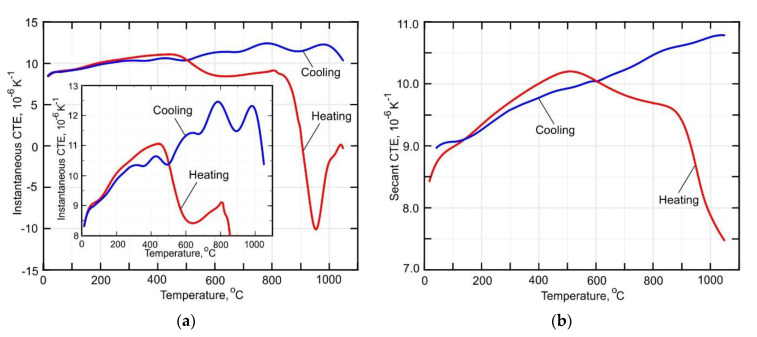
Temperature dependent coefficient of thermal expansion of (**a**) an instantaneous and (**b**) secant.

The experimentally obtained temperature dependence of the thermal strain is shown in Figure 13. It is not difficult to see that the heating and cooling parts of the curve have different slopes for temperatures above 600 °C. The temperature dependence of the thermal expansion coefficient was obtained according to the method described in detail in Section 2.4. A decrease of about 20% in the CTE occurs in the temperature range between 400 °C and 600 °C during heating, as seen in Figure 14a. According to [29], this can be explained by the diffusion-controlled phase transformation α′→ α + β, which is accompanied by a slight volume decrease. Note, that above 800 °C, the α + β → β transformation begins and that the transformation rate is not constant. However, the transformation rate is rather slow in the interval between 800–900 °C, which is clearly visible in the secant CTE curve shown in Figure 14b. An increase of the diffusion mobility of the atoms at temperatures above 900 °C leads to a sufficient increase in the rate of β-phase formation. During holding at 1050 °C, the β-phase content reaches 100%, which leads to a reduction of the sample’s volume. However, the coefficient of thermal expansion does not change significantly during cooling.

### 3.5. Analysis of the Stress Relaxation

The instability of the phase composition of the material and the relaxation of the residual stresses arising in the parts due to various technological operations may cause spontaneous changes of their size and shape over time, affecting their service properties. The conditions for relaxation are described by the following equation:

(4) $$\varepsilon_{0}=\varepsilon^{e}+\varepsilon^{p}=\frac{\sigma }{E}+\varepsilon^{p}=const\;\mathrm{at}\;\varepsilon^{e}\neq const;\;\varepsilon^{p}\neq const,$$

where $\varepsilon_{0}$ is the initial total strain, $\varepsilon^{e}$ is the elastic strain, and $\varepsilon^{p}$ is the plastic strain.

**Figure 15:**
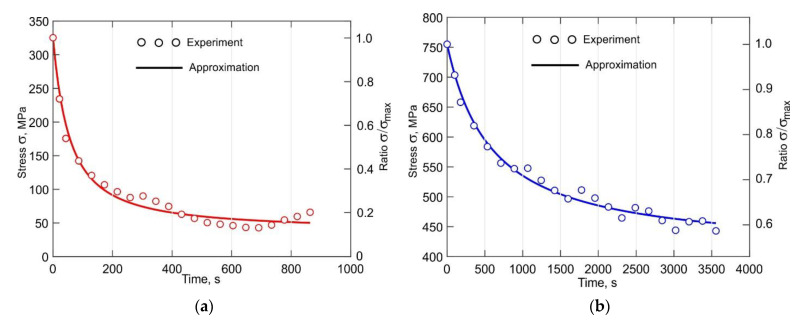
Stress relaxation curves of the DLD-processed Ti-6Al-4V alloy for (**a**) 700 °C and (**b**) 500 °C.

The total strain during the stress relaxation test remains constant due to the increase in plastic strain over time caused by the decrease of the fraction of the elastic strain. These processes can have a considerable effect on the shape stability of the part during DLD as well as during service. The experimentally obtained stress relaxation curves for 500 °C and 700 °C are shown in Figure 15. Note that for clarity, only a small part of the recorded experimental points is shown, as the data recording frequency was 500 Hz. The experimental data were approximated according to the following equation, which describes the intergranular diffusion relaxation processes [57]:

(5) $$\sigma =\sigma_{o}\cdot \exp\left(-\frac{k\cdot t}{1+p\cdot t}\right),$$

where $\sigma_{o}$ is the applied stress, and *k* and *p* are coefficients dependent on the temperature, the microstructure, and the phase composition.

To determine the instantaneous creep strain rate, the time derivative of Equation (5) is obtained:

(6) $$\overset{•}{\varepsilon_{c}}=\frac{d\varepsilon_{c}}{dt}=-\frac{1}{E}\frac{d\sigma }{dt},$$

where *E* is the Young’s modulus at a given temperature.

By differentiating Equation (5) and substituting it into Equation (6) the instantaneous creep strain rate is obtained:

(7) $$\overset{•}{\varepsilon_{c}}=\frac{\sigma_{o}}{E}\cdot \exp\left(-\frac{k\cdot t}{1+p\cdot t}\right)\cdot \left[\frac{k}{{\left(1+p\cdot t\right)}^{2}}\right].$$

**Figure 16:**
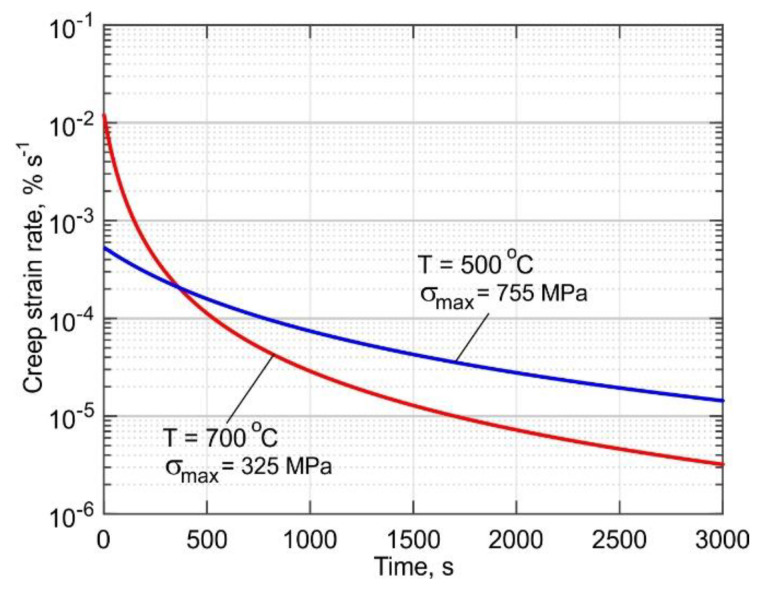
Creep rate curves of the DLD-processed Ti-6Al-4V for 500 °C and 700 °C.

It is a well-known fact that stress relaxation is a thermally activated process, which is particularly effective at high temperatures. Two regions can be distinguished in the curves shown in Figure 15. The first is characterized by an abrupt stress drop, and the second is characterized by a slow stress drop. As noted in [56], the sharp stress decrease at the beginning of the relaxation process is associated with the elimination of a large number of lattice defects. Over time, the amount of lattice distortions decreases, causing the relaxation rate to become slower. In addition, it can as well be explained by the fact that at the beginning of the relaxation phase, the value of the applied stress is high and thus closer to the yield strength of the individual crystallites and mosaic blocks. In the second region, the relaxation curve is asymptotic to a straight line that is parallel to the abscissa axis and shifts from it by the value of the peak stress at which relaxation will not occur. The kinetics of these processes are well illustrated by the creep rate curves shown in Figure 16. The creep rate at 700 °C is 0.12 × 10^−3^ % s^−1^ at the beginning of the relaxation process, which is 23 times higher than the creep rate at 500 °C, as seen in Figure 15a. A 50% reduction of the stress occurs during the first 60 s at 700 °C. After a sharp decrease, the stress continues to decrease, but at a considerably lower rate. In the first 600 s, the stress is reduced by a total of 86% at 700 °C and by 25% at 500 °C, as seen in Figure 15. Hence, it can be concluded that the overheating of the buildup due to its size and/or absence of inter-pass dwell time will lead to a significant reduction of the residual stresses. Therefore, published data on experimentally measured residual stresses without a detailed description of the process parameters affecting the temperature field cannot be used to analyze and verify the accuracy of simulation procedures.

## 4. Conclusions

The mechanical properties of the DLD-processed Ti-6Al-4V alloy were obtained by a tensile test performed in the temperature range between 20 °C and 800 °C. The influence of the thermal history on the stress relaxation for the cases with maximum temperatures of 500 °C and 700 °C were studied. In addition, the temperature dependence of the coefficient of thermal expansion was obtained. The influence of the initial microstructure of the samples on the deformation and the fractures at elevated temperatures was analyzed. An approximation of the measured tensile curves for given temperatures using a proposed fitting function was performed to describe the hardening behavior during plastic deformation. The following conclusions are drawn:

1. The microstructure of the buildup obtained by direct laser deposition with inter-pass temperatures in the range of 60–80 °C consists of a lamellar α’-phase and a small amount of residual β-phase.
2. According to the obtained stress curves, the yield strength decreases gradually by approximately 40% when the temperature increases to 500 °C. Furthermore, it was determined that the softening rate increases significantly upon further heating
3. It was found that the DLD-processed Ti-6Al-4V alloy has a Young’s modulus with greater thermal stability than conventionally processed alloys. At 500 °C, the Young’s modulus of the alloy is about 46% higher than that of the wrought alloy.
4. The analysis of the CTE curves showed that a diffusion-controlled transformation of α’→ α + β in the temperature range between 400 °C and 600 °C leads to a 20% decrease in the CTE. In addition, the α + β → β transformation was determined to start at temperatures above 800 °C.
5. The stress relaxation process was found to have a decisive influence on the formation of the residual stresses at temperatures above 700 °C, which is especially important in the production of small-sized parts by the DLD method.

## Figures and Tables

**Table 1:** Mechanical properties and fitting coefficients.

T, °C	σ_0.2_, MPa	E, GPa	True Tensile Curve			Engineering Tensile Curve		
			*p*_1_	*p*_2_	*p*_3_	*p*_1_	*p*_2_	*p*_3_
20	1000	124.0	0.49292	1.16000	0.012126	−0.34052	1.110000	0.0100000
200	845.0	121.0	0.49292	1.30004	0.012126	−0.34052	1.245515	0.0096846
400	704.8	118.5	0.47779	1.37959	0.009724	−0.33283	1.328941	0.0087338
500	600.0	110.9	0.39231	1.52868	0.007098	−0.64584	1.483647	0.0064257
600	418.8	88.2	−0.69472	1.93472	0.012777	−1.49563	1.842861	0.0115448
700	245	65.0	−1.5021	2.15010	0.018110	−2.46304	1.806981	0.0165517
800	70	26.5	−2.11940	3.33223	0.028902	−2.71973	3.036418	0.0238623
